# Tumor characteristics and survival rate of HER2-low breast cancer patients: a retrospective cohort study

**DOI:** 10.1038/s41598-023-43186-8

**Published:** 2023-10-04

**Authors:** Fereshteh Abbasvandi, Mahdis Bayat, Atieh Akbari, Fatemeh Shojaeian, Ashkan Zandi, Jamal Rahmani, Maryam Omrani Hashemi, Mohammad Esmaeil Akbari

**Affiliations:** 1 Cancer Research Center, Shohadaye Tajrish Hospital, Shahid Beheshti University of Medical Sciences, Tehran, Iran; 2 ATMP Department, Breast Cancer Research Center, Motamed Cancer Institute, ACECR, P.O. Box 1517964311, Tehran, Iran; 3 Department of Surgery, Johns Hopkins University School of Medicine, Baltimore, MD USA; 4 School of Electrical and Computer Engineering, Georgia Institute of Technology, Atlanta, GA 30332 USA

**Keywords:** Oncology, Cancer, Breast cancer

## Abstract

HER2 is an important prognostic marker in breast cancer (BC) patients, which also plays a crucial role in their therapeutic plan. Consequently, a great desire is to thoroughly assess the patients based on their HER2 status. In the current study, we aimed to evaluate HER2-low breast cancer as a new subtype in the standard classification of BC patients and review its characteristics and survival rate in a tertiary center in Iran. We retrospectively evaluated disease-free survival (DFS), overall survival (OS), and clinicopathological characteristics of BC patients referred to the Cancer Research Center in Tehran, Iran from 1991 to 2022. Patients’ clinical characteristics, including HER2 status, which is classified as HER2-low, HER2-positive, or HER2-negative, were obtained from prospectively maintained registries. Among the total 3582 recruited patients, 60.2%, 13.6%, and 26.2% were HER2-negative, HER2-low, and HER2-positive, respectively. HER2-positive patients showed a significantly higher Hazard Ratio (HR) for DFS (HR 1.44, 95% CI 1.01–2.05) and OS (HR 2.05, 95% CI 1.31–3.20), compared to HER2-low. Moreover, HER2-low and HER2-negative were found to show the same proportion of high-grade tumors (28 and 28.4%), while 40% of the HER2-positive tumors were high-grade. Accordingly, HER2-low patients had a lower metastasis risk than the others (*P*-value = 0.01). The Ki67 percentage was significantly lower in the HER2-low group compared to the HER2-positive (*P*-value < 0.001). HER2-low, a new subtype of HER2-status classification with distinct biological and clinicopathological traits, represented the highest survival rate and less invasive characteristics. This difference was statistically significant when compared to HER2-positive, but not when compared to HER2-negative.

Research registration unique identifying number: NCT05754047.

## Introduction

Breast cancer (BC) is the most common malignancy diagnosed in women worldwide, and about 14–20% of the patients are determined as human epidermal growth factor receptor 2 (HER2) positive^1,2^. Human epidermal growth factor receptor 2 (also known as ERBB2) is a membrane protein tyrosine kinase receptor and overexpression of the HER2 gene could lead to worse prognosis by affecting cell proliferation, migration, survival, angiogenesis, tumor invasion, and metastasis. On the other hand, patients could benefit from a HER2-targeted treatment approach^3–6^. While HER2-positive tumors usually show an aggressive clinical course and poor prognosis, in estrogen receptor (ER) and progesterone receptor (PR) negative tumors, being HER2-positive has shown a better prognosis than triple-negative BCs^7^.

There are different HER2-targeted agents, one of which is trastuzumab. Trastuzumab is a monoclonal antibody that targets the extracellular domain of HER2 and alters the normal tyrosine kinase signaling, which is frequently used in clinics. This agent is currently prescribed in combination with chemotherapy in neoadjuvant, adjuvant, and metastatic settings. Other HER2-targeted agents are pertuzumab (humanized monoclonal antibody), tyrosine kinase inhibitors (TKIs, e.g., lapatinib, neratinib, and tucatinib), and antibody–drug combinations (such as TDM-1). These targeted drugs have dramatically changed the prognosis of HER2-positive breast cancer patients over time^8^.

HER2 is currently being assessed by using a combination of immunohistochemistry (IHC) and in situ hybridization (ISH) and could be reported in different states as follows: HER2-negative, HER2 IHC 1 + , HER2 IHC 2 + /ISH-negative, HER2 IHC2 + /ISH-positive, and HER2 IHC 3 + . In recent studies, there is a growing interest in a new classification of breast cancer, termed HER2-low (defined as patients with HER2 IHC1 + and HER2 IHC2 + /ISH-negative in their pathology). This new HER2-low class accounts for more than half of all non-positive HER2 breast cancer patients, so we profoundly need to investigate the prevalence and prognosis of these patients as a distinct group^3–5^. Recent studies are currently showing controversial results about the survival rate of HER2-low BC patients. While some studies did not report any significant differences in disease-free survival (DFS) and overall survival (OS) of different HER2 statuses in metastatic^9–11^ and non-metastatic settings^12,13^, a large cohort study showed better relapse‐free survival in HER2-low BCs than HER2-negative in non-metastatic BCs^14^ and another study indicated decreased DFS and OS in HER2 ≥ IHC 1 + ^15^.

Owing to the high prevalence of breast cancer all over the world, it seems crucial to dive deeply into this distinct group of patients to understand the clinical and molecular pathology features of different HER2 statuses. Accordingly, the current study is designed to assess the DFS, OS, and clinicopathological features of breast tumors based on HER2 status among patients referred to a cancer research center in Tehran, Iran.

## Material and method

### Study design

**Figure 1:**
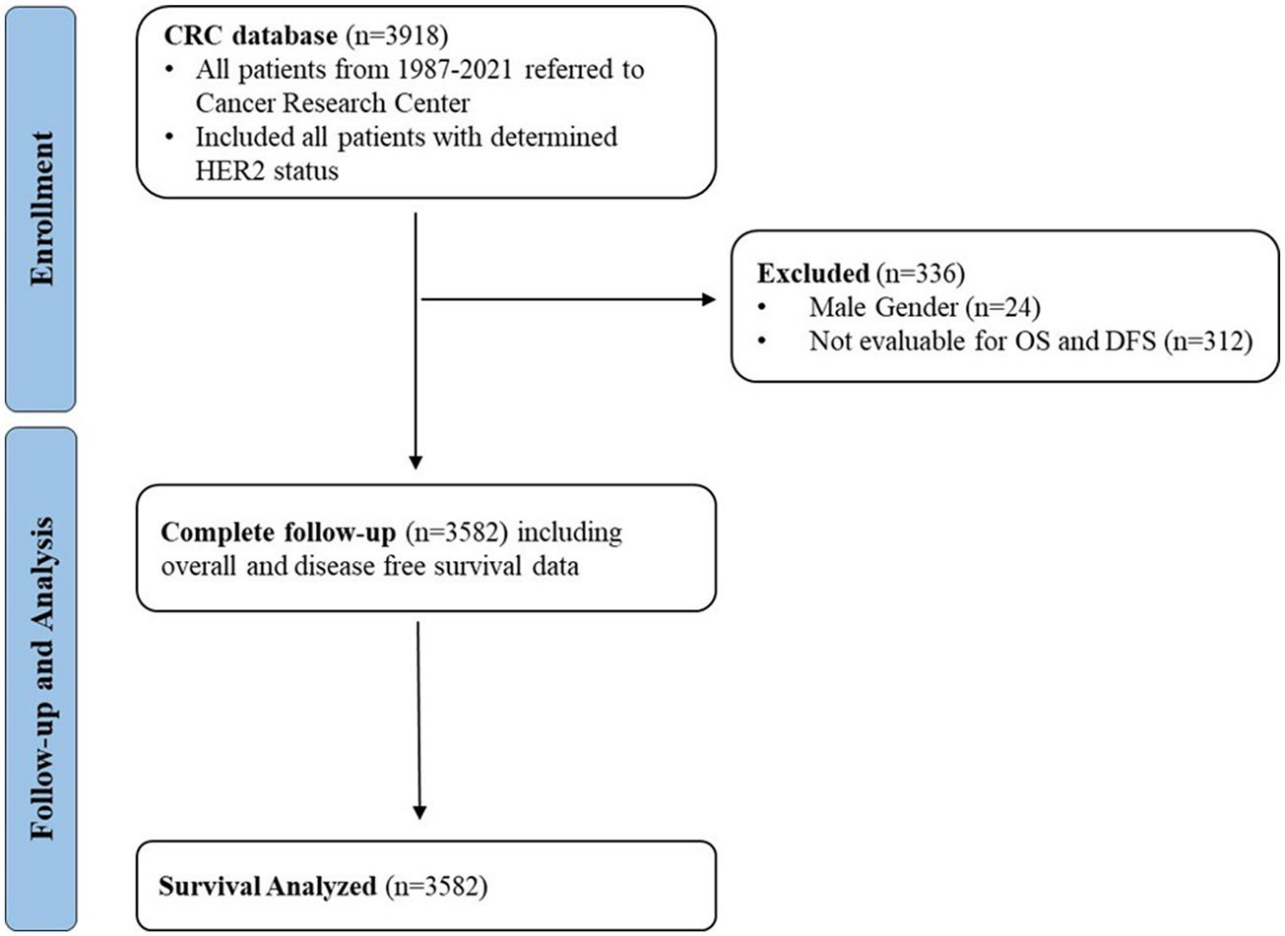
Flow diagram of the study.

Patients diagnosed with breast cancer between April 1991 and March 2022 were identified from prospectively maintained breast cancer registries in the Cancer Research Center (CRC) in Tehran, Iran. All the patients who were referred to the CRC from April 1991 to March 2022 who underwent surgery were included in the study and their diagnoses were verified by an experienced breast pathologist at the center through histopathological examination. From a total of 3918 patients the following patients were excluded (Fig. 1):

1. Male patients
2. Patients with indeterminate or missing HER2 status
3. Patients with missing DFS and OS information (zero encounter/follow-up)

The study was approved by the Shahid Beheshti University of Medical Sciences and the respective ethics committees in the participating institution and all methods were performed in accordance with the relevant guidelines and regulations, also, patient information confidentiality was upheld (ethics number: IR.SBMU.CRC.REC.1401.034). The work has been reported in line with the STROCSS criteria^16^. The informed consent was obtained from all patients.

### Variables and outcome measure

Demographic information (including age, gender, and family history), tumor characteristics (including estrogen receptor (ER), progesterone receptor (PR), Ki67, tumor grade, stage, HER2 IHC, and HER2 ISH status), and treatment plan (determining if the patients received chemotherapy, hormone therapy, and trastuzumab (Herceptin)) were obtained from the hospital information system. The stage of the tumor was determined using the TNM staging system of the breast cancer^17^.

The patients in this study received treatment in accordance with the latest standard guidelines for chemotherapy and radiotherapy, regardless of their HER2 status. Every patient in the study underwent breast surgery, which included either mastectomy or breast-conserving surgery. Additionally, all patients identified as HER2-positive received Herceptin, while none of those with HER2-negative or HER2-low status were administered Herceptin. Basic information about the patients is presented in Table 1 in detail. Patients are undergoing regular follow-up at the cancer research center through physician visits at clinics or maintaining contact with patients via phone. Any instances of cancer recurrence or mortality have been diligently documented, including the date and cause of events, and cancer-related deaths were included in the survival analysis. Cancer recurrence sites were also obtained up to 15 years of follow-up.

**Table 1:** Baseline patients characteristics.

Variables	HER2 categories			*P*-value
	HER2-negative n = 2155 (60.2%)	HER2-low n = 489 (13.6%)	HER2-positive n = 938 (26.2%)	
Age (years)	49.26 ± 11.80	49.15 ± 11.31	48.01 ± 10.97	0.02
Ki67%	25.72 ± 22.49	22.27 ± 21.72	30.40 ± 19.76	0.01
ER status				0.01
Positive	1607 (74.6)	420 (85.9)	564 (60.1)	
Negative	543 (25.2)	67 (13.7)	359 (38.3)	
Unknown	5 (0.2)	2 (0.4)	15 (1.6)	
PR status				0.01
Positive	1510 (70.1)	394 (80.6)	469 (50.0)	
Negative	642 (29.8)	93 (19.0)	457 (48.7)	
Unknown	3 (0.1)	2 (0.4)	12 (1.3)	
Herceptin (No)	2154 (99.9)	488 (99.8)	522 (55.7)	0.01
Hormone therapy (No)	382 (17.7)	46 (9.4)	275 (29.3)	0.01
Family history (No)	1321 (61.3)	264 (54.0)	550 (58.6)	0.04
Surgery				0.01
BCS	1520 (70.5%)	364 (74.4%)	559 (59.6%)	
Mastectomy	517 (24%)	109 (22.3%)	339 (36.1%)	
Unknown	118 (5.5%)	16 (3.3%)	40 (4.3%)	
Chemotherapy (No)	237 (11.0)	60 (12.3)	47 (5.0)	0.01
Recurrence/metastasis				0.01
No recurrence/metastasis	1847 (85.8)	436 (89.2)	753 (80.3)	
Locoregional recurrence	89 (4.1)	19 (3.9)	49 (5.2)	
Bone metastasis	57 (2.6)	11 (2.2)	30 (3.2)	
Visceral and other sites	162 (7.5)	23 (4.7)	106 (11.3)	
Stage				0.01
0	3 (0.1)	1 (0.2)	1 (0.1)	
1	440 (20.4)	113 (23.1)	112 (11.9)	
2	909 (42.2)	220 (45.0)	377 (40.2)	
3	642 (29.8)	126 (25.8)	368 (39.2)	
4	46 (2.1)	4 (0.8)	20 (2.1)	
Unknown	115 (5.4)	25 (5.1)	60 (6.5)	
Grade				0.01
1	233 (10.8)	52 (10.6)	40 (4.3)	
2	1089 (50.5)	254 (51.9)	394 (42.0)	
3	611 (28.4)	137 (28.0)	381 (40.6)	
Unknown	222 (10.3)	46 (9.5)	123 (13.1)	

Patient HER2 status was determined using immunohistochemistry staining (IHC) and categorized as 0, + 1, + 2, or + 3. Specifically, scores of 0, + 1, and + 3 correspond to HER2-negative, HER2-Low, and HER2-positive, respectively. Patients with a HER2 score of + 2 underwent further evaluation via in situ hybridization (ISH). Subsequent ISH analysis provides a more accurate classification of HER2 expression as positive or negative; patients with positive ISH results are considered HER2-positive, while those with negative ISH results are categorized as HER2-Low. In summary, HER2-negative was defined as the IHC score of 0, HER2-positive was defined as the IHC score of 3 + or IHC score of 2 + /ISH-positive, while patients with IHC score of 1 + or 2 + /ISH-negative were defined as HER2-low.

### Statistical analysis

Continuous and categorical variables were reported as mean ± standard deviation (SD) and frequency counts (percentages), respectively. Continuous and categorical variables were compared using analyses of variance (ANOVA) and χ2 tests. Kaplan–Meier analysis was used for univariate analysis, and log-rank tests were used for group comparisons. Cox proportional hazards regression was used for multivariate analysis (DFS adjusted for age of diagnosis, family history, Herceptin usage, ER status, PR status, and grade of cancer and OS adjusted for age of diagnosis, family history, Herceptin use, ER status, PR status, grade, and recurrence state). Using scaled Schoenfeld residuals, the assumption of proportional hazard was checked for the final models. IBM SPSS 23.0 and STATA 14 were used for statistical analyses, and a P-value < 0.05 was considered statistically significant.

### Ethical approval

This study got the code number from the ethics committee, which was supported by the deputy of research and technology at Shahid Beheshti University of Medical Sciences (ethics number: IR.SBMU.CRC.REC.1401.034).

## Results

### Patients’ characteristics

Figure 1 shows the flow diagram of the included patients. There were 3918 patients with human epidermal growth factor receptor 2 (HER2) status in the database of the Cancer Research Center (CRC), among which, 24 male patients and 312 patients with missing DFS and OS information and no encounter after the first visit, were excluded. In total, 3582 patients have been included in the DFS and OS analysis.

Table 1 provides the patients' baseline characteristics across the HER2 status. Among included patients, 60.2%, 13.6%, and 26.2% of patients were HER2-negative, HER2-low, and HER2-positive, respectively. The differences in the patient mean age were significant between the groups (*P*-value = 0.02), and the HER2-negative group with a mean age of 49.26, was the oldest one. The frequency of positive ER and PR was more than negative among all the groups; however, the HER2-low group had the highest proportion of ER and PR positive status. Regarding Ki67, the HER2-low group had the lowest Ki67 among the whole groups (22.27 ± 21.72), although the difference wasn’t significant between HER2-low and HER2-negative (*P*-value = 0.14), it was significant among the HER2-low and HER2-positive (*P*-value < 0.001).

**Figure 2:**
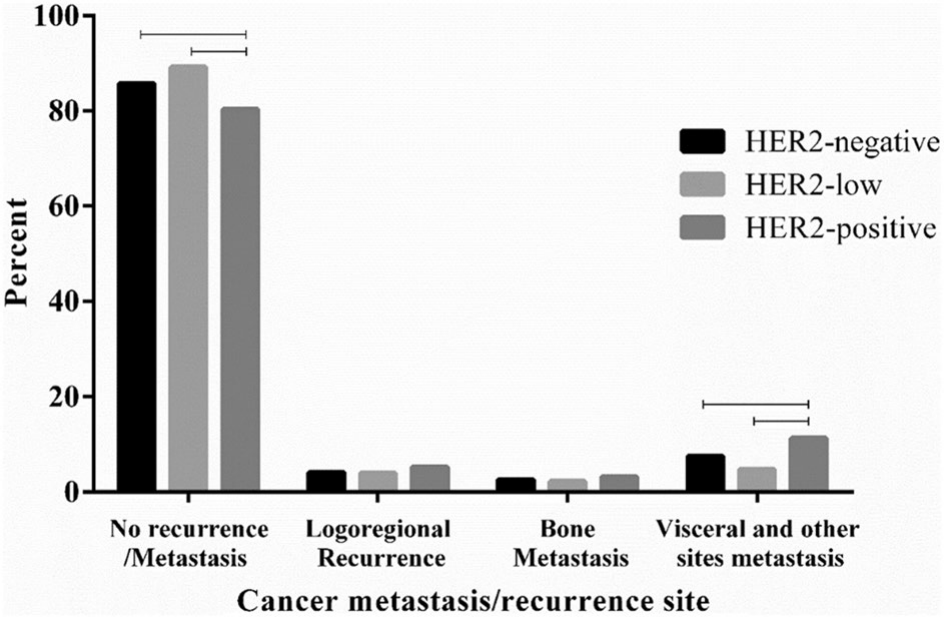
Cancer recurrence/metastasis site according to HER2 categories.

Distribution of cancer recurrence/metastasis site according to HER2 categories provided in Fig. 2. In the no recurrence/metastasis group, HER2-negative and HER2-low patients were significantly more than HER2-positive patients (*P*-value < 0.001). In the bargain, among the patients with visceral and other metastasis sites (other than bone and locoregional), HER2-positive patients were significantly more than HER2-negative (*P*-value = 0.002) and HER2-low patients (*P*-value < 0.001). Furthermore, in the no recurrence/metastasis group, HER2-low patients contain the majority of the patients (*P*-value = 0.01), while in visceral and other sites metastasis HER2-positive patients are the most (*P*-value < 0.001).

### Free-disease survival outcomes

**Figure 3:**
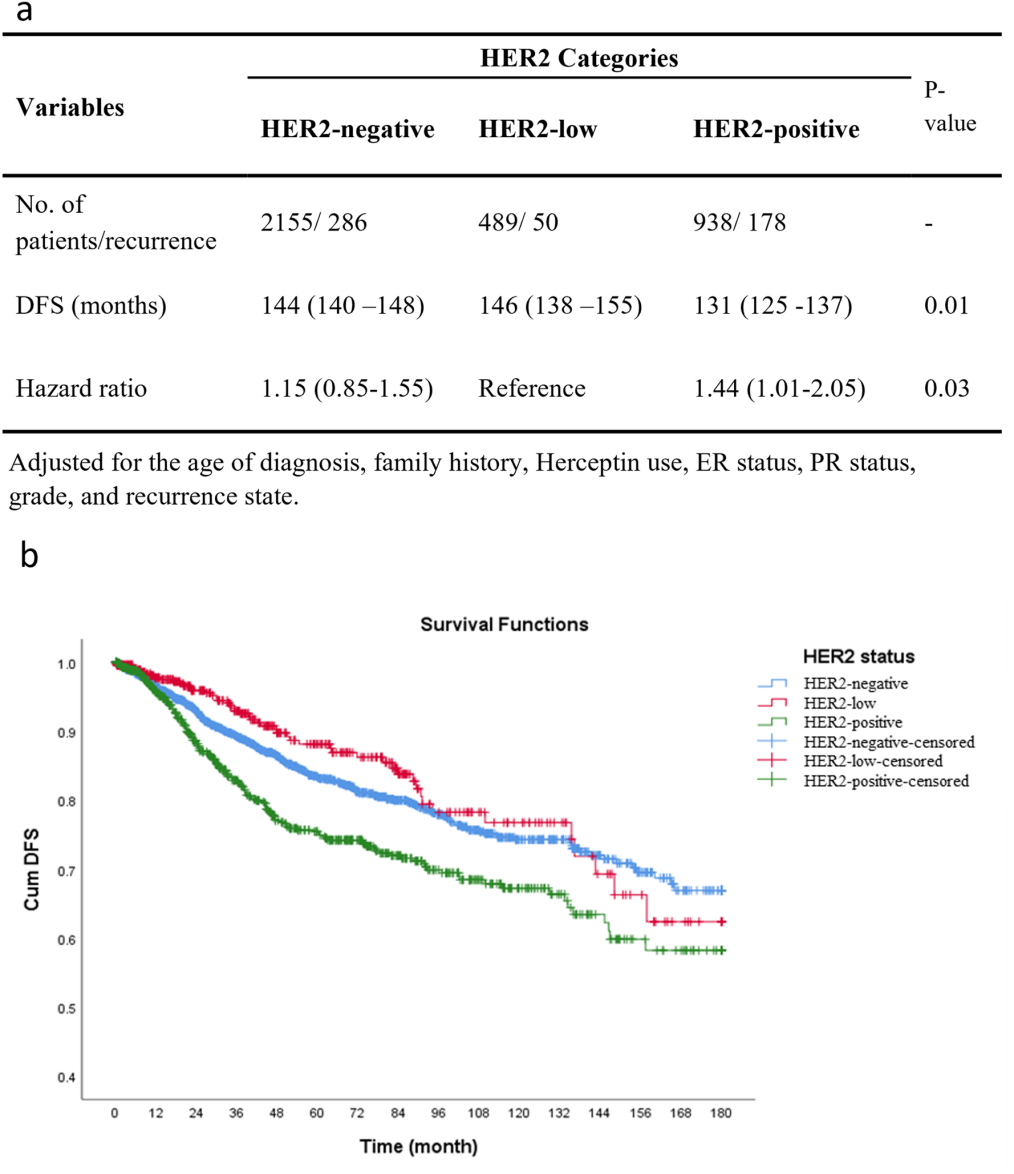
(**a**) Disease-free survival for different HER2 status. (**b**) Kaplan–Meier curves of disease-free survival (DFS) among patients according to HER2 status (*P*-value < 0.001, of Log-rank test).

The median follow-up of 3582 patients included in the DFS analysis was 33 (IQR 10–76) (mean 48.8; 95% CI 47.3–50.4) months. Altogether, 514 (14.3%) DFS events were observed among patients. HER2-low had the highest mean of DFS (146 months), while HER2-positive had the lowest duration of DFS (131 months) (Fig. 3a). In all HER2 statuses, the 5-year, 10-year, and 15-year DFS rates were 80%, 73%, and 64%, respectively. In detail, the mentioned DFS rates, in the HER2-negative, were 81%, 75%, and 67%, respectively, in the HER2-low group they were 87%, 78%, and 63%, respectively, and they were 74%, 66%, and 58%, in the HER2-positive patients (Fig. 3b). Compared to HER2-low group patients, HER2-positive patients had a significantly higher Hazar Ratio (HR) for DFS (HR 1.44, 95% CI 1.01–2.05); however, HER2-negative patients showed no significant HR difference for DFS compared to the HER2-low.

### Overall survival outcomes

**Figure 4:**
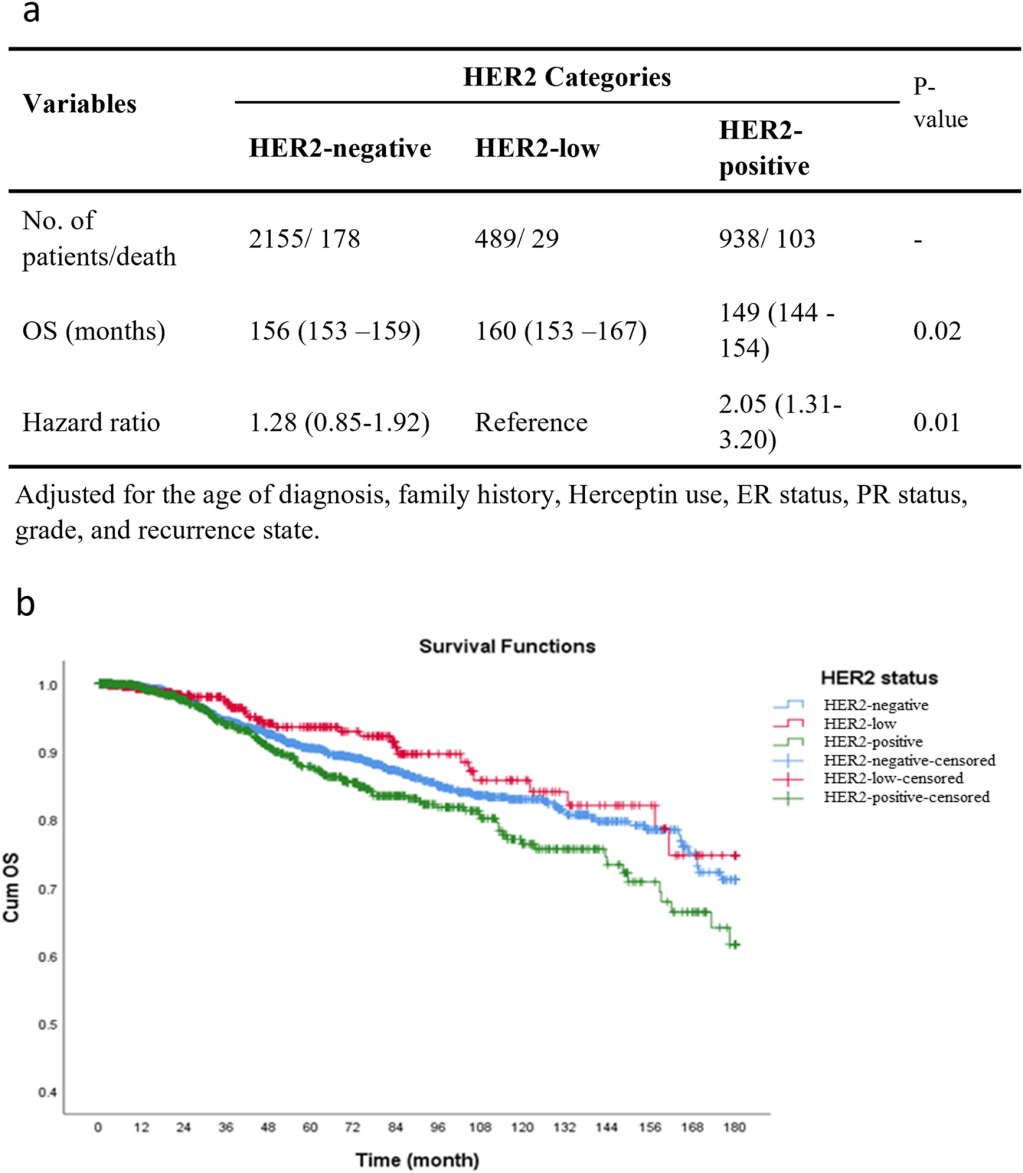
(**a**) Overall survival of the patients based on HER2. (**b**) Kaplan–Meier curves of overall survival (OS) among patients according to HER2 status (*P*-value of Log-rank test = 0.01).

The median follow-up of 3582 patients included in the OS analysis was 38 (IQR 12–81) (mean 52.5; 95% CI 50.9–54.1) months. During follow-up, 310 (8.7%) deaths occurred among patients. The HER2-low had the highest mean of OS at 160 months, and the HER2-positive had the lowest duration of OS at 149 months, and a P-value of 0.02 shows the significance of this difference (Fig. 4a). The 5-year, 10-year, and 15-year OS rates of all patients were 89%, 80%, and 69%, respectively. To be more precise, in the HER2-negative group, it was 89%, 81%, and 71%, respectively, in the HER2-low group it was 93%, 84%, and 74%, respectively, and it was 85%, 76%, and 62%, in the HER2-positive patients (Fig. 4b). Compared to HER2-low group patients, although HER2-positive patients had significantly higher HR for OS (HR 2.05, 95% CI 1.31–3.20), HER2-negative patients showed no significant difference for OS compared to HER2-low.

### HER2-low subgroup analysis

The HER2-low group contained 401 participants with HER2 IHC 1 + and 88 participants with HER2 IHC 2 + /ISH-negative status. While HER2 IHC 1 + showed no significant difference in HR of DFS (HR = 0.99, 95% CI 0.73–1.35) compared to HER2-negative, HER2 IHC2 + /ISH-BC had significantly lower HR for DFS (HR = 0.12, 95% CI 0.02–0.84) compared to HER2-negative group (Table 2). HER2 IHC 1 + did not show any significant HR difference, compared to HER2-negative participants. In addition, results show no significant difference for the HER2 IHC 1 + group compared to the HER2-negative group in HR of mortality (HR = 0.88, 95% CI 0.59–1.32).

**Table 2:** Disease-free survival and overall survival for different HER2 status (HER2-low subgroups).

Variables	HER2 categories				*P*-value
	HER2-negative	HER2-low		HER2-positive	
		IHC1 +	IHC2 + /ISH negative		
No. of patients/recurrence/death	2155/286/178	401/49/29	88/1/0	938/178/103	–
DFS Hazard ratio	Reference	0.99 (0.73 –1.35)	0.12 (0.02–0.84)	1.25 (0.98 -1.60)	0.20
OS Hazard ratio	Reference	0.88 (0.59 –1.32)	^-^*	1.60 (1.21–2.12)	0.01

Adjusted for the age of diagnosis, family history, Herceptin use, ER status, PR status, and grade of cancer. The hazard ratio of death adjusted for the above confounders and recurrence state.

*No statistics are computed because all cases are censored.

### Multivariate analysis

Multivariate analysis for included variables in DFS and OS analysis is provided in Table 3. In DFS multivariate analysis, patients with grades 2 and 3 had higher HR for DFS, 1.39 (95% CI 0.92–2.11) and 1.68 (95% CI 1.10–2.57), compared to patients with grade 1 tumor. Multivariate analysis of OS outcome showed that the age of breast cancer diagnosis is a significant factor in OS and patients who have been diagnosed with breast cancer at an age equal to or more than 50 years had higher HR for death (HR = 1.49, 95% CI 1.18–1.87). Although the overall survival was not significantly different among the tumor grades, the higher tumor stage (stage 3–4), significantly affected the overall survival (HR = 5.92, 95% CI 3.58–9.79). In addition, ER-positive patients showed better survival compared to ER-negative patients (HR = 0.66, 95% CI 0.44–0.99), and patients with recurrence had a higher hazard ratio for mortality compared to patients with no recurrence (HR = 1.96, 95% CI 1.36–2.84).

**Table 3:** Multivariate analysis of patients’ characteristics, hazar ratio of their disease-free survival, and overall survival.

Variables	Multivariate analysis, HR (95% CI)	
	Disease-free survival	Overall survival
Age		
< 50	1	1
≥ 50	0.92 (0.76–1.10)	1.49 (1.18–1.87)
PR		
Negative	1	1
Positive	0.77 (0.58–1.03)	1.21 (0.81–1.80)
ER		
Negative	1	1
Positive	0.86 (0.62–1.19)	0.66 (0.44–0.99)
Family history		
No	1	1
Yes	0.87 (0.70–1.08)	1.06 (0.81–1.39)
Grade		
1	1	1
2	1.39 (0.92–2.11)	1.47 (0.84–2.57)
3	1.68 (1.10–2.57)	1.73 (0.98–3.05)
Stage		
0–1	1	1
2	1.19 (0.84–1.69)	1.64 (0.98–2.74)
3–4	3.49 (2.48 -4.90)	5.92 (3.58–9.79)
Hormone therapy		
No	1	1
Yes	1.03 (0.75–1.41)	0.78 (0.52–1.16)
Chemotherapy		
No	1	1
Yes	1.35 (1.76–2.37)	1.82 (0.73–4.51)
Recurrence		
No	–	1
Yes	–	1.96 (1.36–2.84)

Among 938 HER2-positive patients, 412 of them were administered Herceptin. Multivariate analysis showed significantly lower mortality among Herceptin users compared to non-users (HR = 0.51, 95% CI 0.32–0.80). However, Herceptin administration among HER2-positive participants showed no significant difference in DFS compared to non-users (HR = 1.14, 95% CI 0.83–1.56).

## Discussion

In the current large-scale cohort study, we have assessed the epidemiological and survival characteristics of HER2-low breast cancer patients, along with HER2-negative and HER2-positive groups in a total of 3582 patients. Taken altogether, disease-free and overall survival rates were better among HER2-low patients compared to other groups; this difference was significant between the HER2-low and HER2-positives ones. This finding would lead us to the idea that this new HER2-low group could be a distinct biological subtype with unique clinicopathological traits and needs to come into consideration as an additional subtype to the classical classification of the HER2-positive vs. negative and would expand the traditional subgroups of HER2 expression in breast cancer patients.

The total proportion of HER2-positive tumors among the 3582 patients in our study was 26.2%; in addition, about 13.6% of the patients were HER2-low. Taken together, 39.8% of the patients in this cohort showed at least some level of HER2 expression. However, other studies have shown a higher percentage of the HER2-low subgroup, which contained about 30–50% of the whole study population^18–20^. This observed difference could be due to different sampling or sample sizes, as well as possible variations in the genetic background of patients in different countries.

Consistent with the previous studies, our cohort showed a significantly higher frequency of hormone receptor (ER and PR) positivity in the HER2-low group compared to the others^4,9,14,21,22^. In line with this observation, in another study, HER2-low tumors were also found in much greater numbers among hormone receptor-positive breast cancer than HER2-negative tumors^20^. In terms of grade, in HER2-low and HER2-negative patients, about 10% were in grade I, while only 4.3% were low grade in HER2-positive patients. Hence, although a significant difference has been seen between HER2-positive and the rest, the HER2-negative and HER2-low showed the same proportion of low-grade tumors, which is in contrast with the result of another research that showed the lower grade in HER2-low patients compared to HER2-negative group^20^. Regarding the stage of the tumor, about 2.1% of HER2-positive and HER2-negative patients were in stage 4 at the time of diagnosis, while only 0.8% of the HER2-low patients were at the same stage. The tumor of HER2-low patients had the lowest mean of ki67 among the patients and this difference was significant between HER2-low and HER2-positive groups. Taken altogether, it seems that this new subgroup is showing less aggressive behavior regarding the clinicopathological characteristics and is more similar to the HER2-negative group rather than the HER2-positive.

In our study, HER2-low patients showed the highest DFS and OS (146 and 160 months, respectively) among the whole patients. Although the differences were significant between HER2-low and HER2-positive groups, they were not statistically significant between HER2-low and HER2-negative patients. This is consistent with another study from Korea, in which they have not reported any significant difference in overall survival between the HER2-low and HER2-negative BCs, in line with a couple of other studies from France, Italy, etc.^9,12,23–25^. However, a recent large multicentral cohort within the Asian Breast Cancer Cooperative Group (n = 28,280), reported a better relapse-free survival rate in HER2-low BCs, rather than in HER2-negative BCs in their multivariable analysis^14^. Consistent with the result of the aforementioned study, some other studies have also presented that HER2-low patients have a significantly longer survival rate than HER2-negative patients^20,22^. All in all, there have been different reports regarding the survival rate of HER2-low patients, ours showed significantly better DFS and OS rates of HER2-low compared to HER2-positive patients, while no significant difference was noticed in HER2-negative and HER2-low groups. Therefore, the HER2-low expression group seems to be more similar to HER2-negative compared to HER2-positive in the current survey.

Taking the genetic background of the patients into consideration, the Mutai et al. study reported markedly improved outcomes for HER2-low expression compared to HER2-negative in women with high genomic risk^26^. So different genetic backgrounds could be a possible explanation for these controversial reports in the literature. One of the limitations of the current study is the lack of genetic background study, which might be the possible reason for differences in survival rate in this study. In addition, considering different ways of subdividing the patients, Li et al. delineated that patients with HER2-low BCs survived significantly longer than those with HER2-negative BCs in the overall population and HR-positive subgroup, but not in their HR-negative subgroup^21^; So, one other possible explanation for differences in survival rates could be the reference group in which we are comparing the patients.

To dive more deeply into this new HER2 expression subgroup, an important novel finding of our study was that within the HER2-low subgroups, HER2 IHC1 + tumors appeared prognostically distinct from IHC2 + /ISH-negative tumors. While HER2 IHC2 + /ISH-negative BC had significantly better DFS compared to HER2-negative, HER2 IHC1 + did not present a significant difference in DFS and was more like the HER2-negative group. This result would partly contradict previous findings by Ignatov et al. (n = 5907) and Rossi et al. (n = 1150) studies, in which patients with HER2 IHC2 + /ISH-negative and early-stage BC had worse DFS than those with HER2 IHC 0 or 1 + ^27,28^. These differences might be due to variations in patient selection, sample sizes, and follow-up duration for DFS rates, as our study includes all breast cancer patients, not just early-stage ones. In addition, Gilcrease et al., presented that the HER2 IHC1 + subgroup was significantly associated with decreased disease-specific survival, DFS, and OS, and in their cohort, and HER2-negative had a better outcome^15^. This different observation could be explained by the fact that the mentioned study just took the HER2 IHC 1 + subgroup of the HER2-low into consideration. On the whole, this new HER2-low expression group might divide into more subgroups in the near future, considering different survival patterns and clinicopathological characteristics that were observed in different studies.

As previously mentioned, HER2 increased the proliferation and survival of the primary tumor and distant lesions which upon completion of full transformation, caused metastases^29^. In the present study, we found out that the HER2-positive group had a significantly higher rate of visceral and other (than locoregional and bone metastasis) metastasis sites; consistently, in no recurrence/metastasis category, the HER2-positive group was significantly lower than the HER2-negative and HER2-low participants. In the HER2-low subgroup, the metastasis rate was significantly lower than the HER2-negative and HER2-positive BCs. Consequently, the HER2-positive group showed a higher risk of metastasis, especially in other sites (than bone and locoregional) and the HER2-low group showed a lower risk of metastasis compared to the other two groups. Related to metastasis, Guven et al., reported that hormone receptor-positive HER2-low BCs have an increased risk of brain metastasis and inferior DFS compared to hormone receptor-positive HER2-negative^30^, however, in our study, this difference between HER2-low and HER2-negative was not significant.

Taken altogether, the distinction between HER2-low, HER2-positive, and HER2-negative tumors can be made by immunohistochemical evaluation in clinical practice, and this is the most crucial message for the future of breast cancer diagnosis. The hormone receptor-positive, HER2-negative, HER2-positive, and triple-negative breast cancer subtypes have served as the foundation of the biological model of breast cancer for a long time. Our data lends credence to the idea that there are more clinically significant subgroups of breast cancer. These novel subtypes of breast cancer can be identified through standard pathological evaluation of HER2 expression. The molecular landscape of breast cancer subtypes will become much more complex as a result of this strategy, and it will also present new, tailored therapy possibilities for enhancing the prognosis of breast cancer.

The limitations identified in this study, including missing patient information, lack of precise records regarding chemotherapy regimens and response to therapy, absence of genetic background data, reliance on a single-center approach, and inconsistencies in HER2 scoring, collectively have the potential to impact the study's outcomes and implications. For instance, the absence of complete patient information may lead to potential selection biases and result in a reduced sample size, affecting the overall robustness of the findings. The lack of detailed records regarding chemotherapy regimens and response rates, as well as genetic background data, may introduce confounding variables that hinder a comprehensive understanding of chemotherapy regimens and treatment effects on HER2-Low breast cancer patients and potential genetic influences on treatment response and disease progression. The utilization of a single-center study design raises concerns about the diversity of patient populations, treatment practices, and healthcare settings, limiting the generalizability of the results. Taken together, these limitations underscore the importance of interpreting the study's findings with caution and considering their potential influence on the conclusions and recommendations. Addressing these limitations in future research, such as through prospective multicenter studies with genetic information can enhance the readers' understanding of the study's scope and applicability.

## Conclusion

In conclusion, the current study comprised more than 3500 women with different stages of breast cancer from a cancer center in Tehran. The results presented a new subgroup of HER2 expression, HER2-low, which showed distinct survival and clinicopathological characteristics. It showed a significantly better survival rate and less aggressive traits compared to the HER2-positive participants, with higher expression levels of hormone receptors.

## Data Availability

The datasets used and/or analyzed during the current study are available from the corresponding author upon reasonable request.
